# Prevalence of Text Neck Syndrome Among Medical Students in Chennai, India: A Cross-Sectional Study

**DOI:** 10.7759/cureus.100655

**Published:** 2026-01-03

**Authors:** Benilsha J. E., Durga Devi G., Rahe Rajan, Renuka Devi M. R., Devaki P. R.

**Affiliations:** 1 Anatomy, Sree Balaji Medical College and Hospital, Chennai, IND; 2 Physiology, Sree Balaji Medical College and Hospital, Chennai, IND

**Keywords:** ergonomics, mobile phone usage, musculoskeletal disorders, neck disability, neck pain

## Abstract

Introduction: Bad head postures while using mobile phones or other electronic gadgets may eventually cause text neck syndrome. If not treated, this could lead to stress injury and nerve damage. It can also lead to chronic pain in the neck and shoulder, headaches, and long-term problems in the alignment of the spine if left uncorrected.

Aim: To assess the prevalence of text neck syndrome in medical students and to identify the factors associated with it.

Methodology: A cross-sectional study was conducted in the anatomy department of Sree Balaji Medical College and Hospital situated in Chennai, Tamil Nadu, from January to March 2025. The study was conducted among 303 undergraduate medical students studying in the second, pre-final, and final years, selected using a purposive sampling method. A pre-tested structured questionnaire collected data, and the Neck Disability Index (NDI) was used to assess the severity of neck disability. Data was collected and entered into an Excel data sheet (Microsoft Corp., Redmond, WA, USA) and analysed by using IBM SPSS Statistics for Windows, Version 26 (Released 2018; IBM Corp., Armonk, New York, United States).

Results: The mean age of the study participants was 21 ± 2.2. About 167 (56%) were males, and 136 (44%) were females. From the NDI questionnaire, the self-rated disability due to neck pain was found to be 105 (34.7%) with mild disability, 93 (30.70%) with moderate disability, and 23 (7.6%) with severe disability. This analysis showed a statistically significant association, P < 0.05, between the variable lack of awareness, P = 0.006, and the text neck syndrome. The chi-square test was used as the statistical method for this analysis.

Conclusion: The prevalence of undergraduate medical students presenting with text neck syndrome was very high, with the majority showing mild to moderate neck disability. Lack of awareness about posture and lack of ergonomic practices are significantly associated with text neck syndrome. Early implementation of an awareness program and preventive ergonomic interventions will help in reducing long-term musculoskeletal complications.

## Introduction

In the evolving era of modernization and urbanization, the usage of electronic gadgets has become an everyday norm in our lives [1]. Globally, it has been found that 87% of the young population (age group of 14-18 years) and 79% of teenage children (age group of 12 to 15 years) use mobile phones [2]. With education becoming more and more digitalized, prolonged use of electronic gadgets like mobile phones, computers, and tablets could lead to repetitive stress injury, which, left untreated, could lead to permanent nerve damage and spinal injuries. This condition is called text neck syndrome [1]. This is caused due to prolonged usage of mobile phones, with improper bending of the head and neck leading to neck pain [3]. The complications which could occur if left untreated include, compression of spinal discs, spinal disc degeneration, disc herniation, nerve and muscle damage [4]. Globally, the prevalence of text neck syndrome was found to be 44.8% in Pakistan, 35% in the USA, and 41.8% in Malaysia [3-5]. The prevalence of text neck syndrome in India ranges from 25% to around 47% [6-8].

The neck is a biomechanically complex region that consists of cervical vertebrae, intervertebral discs, facet joints, ligaments, muscles, and neural elements that work together to maintain head posture and mobility [9]. Prolonged mobile phone users commonly adopt a sustained forward head posture, which substantially increases the mechanical load on the cervical spine. Studies have implied that progressive neck flexion gradually increases compressive and shear forces across cervical intervertebral discs and facet joints, while there is a corresponding increase in tensile strain on posterior cervical musculature and ligaments [8]. Chronic exposure to such abnormal loading patterns can lead to muscular imbalance, decreased endurance of deep cervical flexors, altered neuromuscular control, and degenerative changes over time [7]. Besides localized neck pain, these biomechanical changes have also been linked to cervicogenic headache, shoulder and upper back pain, as well as postural deformity, including increased thoracic kyphosis. Medical students often represent early and prolonged users of mobile devices because of the academic demands and can thus be at an increased risk of developing text neck syndrome, with its potential to predispose to chronic musculoskeletal symptoms and postural dysfunction later in life [10].

To prevent text neck syndrome, it is crucial to maintain proper ergonomics, take frequent breaks, and perform neck exercises regularly. Students are seldom aware of the long-term effects of gadget usage, leading to morbidity related to musculoskeletal disorders. It can also compromise the overall quality of life of the individual, leading to a decrease in their productivity, poor academics, and concentration difficulties [11]. With this background, the study was conducted among undergraduate medical students in a tertiary care medical college in Chengalpattu district, Tamil Nadu, with an aim of finding out the prevalence of text neck syndrome and the factors associated with its occurrence.

## Materials and methods

A cross-sectional study was conducted in the anatomy department of Sree Balaji Medical College and Hospital situated in Chennai, Tamil Nadu, from January to March 2025. Ethical approval was obtained from the Institutional Ethics Committee of Sree Balaji Medical College and Hospital (Ref. no: 002/SBMCH/IHEC/2024/2309). Before enrolling the medical students in the study, consent was obtained.

Medical students studying in the second, pre-final, and final years using smartphones and other electronic devices were included. Students who have pre-existing neck problems or a history of any recent head/neck injury were excluded.

Sample size

A study done by Alsiwed et al. found the prevalence of text neck syndrome to be 68% among medical students [12]. Using this prevalence (P) in the formula 4PQ/L^2^, a minimum required sample size of 242 was obtained by assuming an allowable error of 6%, considering feasibility, available study duration, and the available undergraduate student population. To increase precision, by allowing for non-response, 303 students were ultimately included using a purposive sampling method. A total of 303 students provided consent and participated in the study.

Study tool

Demographic data were collected, and the Neck Disability Index (NDI) questionnaire was distributed to the participants through the institutional learning management system (LMS). The NDI questionnaire consisted of 10 sections, four sections (pain magnitude, headaches, attention, and sleeping) deal with subjective symptoms, whereas the other six sections are related to everyday life activities (reading, occupation, driving, lifting weights, recreation, and physical hygiene). Each section consists of six responses with a scoring scale that ranges from 0 to 5. The total score ranges from 0 to 50. Based upon the total score, they were categorized into no disability (0-4), mild (5-14), moderate (15-24), severe (25-34), and complete disability (>35) [13]. Factors related to the text neck syndrome were duration of mobile phone usage per day and awareness about the mobile phone usage and its impact on the musculoskeletal system, and were assessed using a pretested structured questionnaire, which was developed based on relevant literature. Face validity of the tool was established through expert review, and internal consistency was assessed using Cronbach’s alpha, which demonstrated acceptable reliability [14-16].

Data analysis

The data, which was obtained, was fed into Microsoft Excel (Microsoft Corp., Redmond, WA, USA) and converted and analyzed using IBM SPSS Statistics for Windows, Version 26 (Released 2018; IBM Corp., Armonk, New York, United States). Chi-square test was used to find the association between text neck syndrome and related variables at a confidence level of 95%.

## Results

Approximately 212 (70%) of the study participants were aged 18-19 years, with 167 (56%) males and 136 (44%) females (Table 1).

**Table 1:** Sociodemographic characteristics of the study participants

S. No.	Variable	Frequency	Percentage (%)
1	Age		
	18-19	212	70
	20-21	91	30
2	Gender		
	Male	167	56
	Female	136	44
3	Year of study		
	Second year MBBS	112	37
	Pre-final year MBBS	131	43
	Final year MBBS	60	20

Most participants were in the mild 105 (34.7%) and moderate 93 (30.7%) neck disability categories, while a smaller proportion had no disability 82 (27.1%) and severe disability 23 (7.6%) (Table 2).

**Table 2:** Distribution of participants according to Neck Disability Index (NDI) scores

S. No.	NDI Score	Neck Disability Category	Frequency	Percentage (%)
1	0 to 4	No disability	82	27.1
2	5 to 14	Mild disability	105	34.7
3	15 to 24	Moderate disability	93	30.7
4	25 to 34	Severe disability	23	7.6
5	>35	Complete disability	0	0

Though the association between neck disability and screen time was not statistically significant (P = 0.199), participants with mild and moderate neck disabilities tended to spend more time on their mobile phones, particularly in the four to six hours and more than six hours categories (Table 3).

**Table 3:** Mobile phone usage patterns across different neck disability categories A chi-square test was performed with three degrees of freedom.

Variable			Screen time				P-value
			Less than 2 hours (n = 35)	2-4 hours (n = 91)	4-6 hours (n = 121)	More than 6 hours (n = 56)	
Neck Disability	No disability	Frequency (n)	10	28	31	13	0.199
		Percentage (%)	28.6	30.8	25.2	23.2	
	Mild	Frequency (n)	4	30	47	24	
		Percentage (%)	11.4	33.0	38.3	42.9	
	Moderate	Frequency (n)	17	27	33	16	
		Percentage (%)	48.6	29.7	28.7	28.6	
	Severe	Frequency (n)	4	6	10	3	
		Percentage (%)	11.4	6.6	7.8	5.4	

Awareness of potential musculoskeletal problems caused by mobile phone usage was the highest among participants with mild and moderate neck disability. However, those with severe disability showed a relatively low level of awareness, which may indicate a need for increased education and awareness in this group to help manage and prevent further disability (Table 4).

**Table 4:** Awareness of musculoskeletal problems related to mobile phone use across neck disability categories * P < 0.05 indicates statistical significance at the 95% confidence level; degrees of freedom = 3; chi-square value = 12.46.

Variable			Awareness of Musculoskeletal Problems Due to Mobile Phone Usage by Neck Disability Categories (N = 303)		P-value
			No (n = 96)	Yes (n = 207)	
Neck Disability	No disability	Frequency (n)	29	53	0.0068*
		Percentage (%)	30.2	25.6	
	Mild	Frequency (n)	32	73	
		Percentage (%)	33.3	35.3	
	Moderate	Frequency (n)	23	70	
		Percentage (%)	24.0	33.8	
	Severe	Frequency (n)	12	11	
		Percentage (%)	12.5	5.3	

## Discussion

The emerging day-to-day mandatory use of mobile phones among medical students has led to usage that may contribute to chronic musculoskeletal problems in later stages of life. The prevalence of mild to severe forms of disability according to the NDI questionnaire was found to be 73%. Only 27% of the participants had no disability according to the questionnaire. Similarly high prevalence was observed in a study done by Alsiwed et al. in Saudi Arabia, where 71.02% of the medical students had some form of disability, with 49.5% having mild disability, 16.1% moderate disability, and 2.6% suffering from severe disability [12]. Comparatively lower prevalence was observed in a study done by Weleslassie et al. in Ethiopia (49.2%) [17]. Contrasting findings were observed in a study done by Kamaraj et al. in Puducherry, where the prevalence was found to be only 16.7% [1]. This may be attributed to differences in the sociodemographic characteristics and the instruments used to quantify the varying grades of text neck syndrome.

The present study found that increasing the usage of mobile phones for hours increases the severity of neck disability among the study participants. Similar results were obtained in a study done by Kamaraj et al. in Puducherry and Khan et al. in Pakistan [1,18]. Awareness needs to be created among the medical students, and a minimum break every 20 minutes of mobile phone use has to be advised for the prevention of neck disability, as evident from a study done by Kim and Koo [19].

The present study found a statistically significant association between lack of awareness regarding musculoskeletal problems, especially the text neck syndrome associated with mobile phone usage. Similar findings were observed in a study done by Varshney et al. and Acharya et al., in which lack of awareness about the health hazards of mobile phone usage was associated with musculoskeletal problems [20,21].

To reduce the prevalence of text neck syndrome among students, it is essential to promote awareness of correct posture during mobile device use and to encourage simple ergonomic practices, such as holding devices at eye level or using stands to minimize neck flexion [22]. Regular breaks from screen use and incorporation of stretching and strengthening exercises for the neck and upper back into the daily routines can further reduce the risk. The Pomodoro technique may be adopted as a practical preventive strategy, involving 25 minutes of focused study followed by a five-minute break. Such scheduled breaks allow posture correction and neck mobility, potentially reducing sustained cervical strain. The duration of study and break intervals can be varied according to individual comfort and academic demands [23].

Educational institutions have a role in sensitizing students through health education programs that emphasize the importance of digital hygiene and the need to limit prolonged and uninterrupted mobile phone usage. Extensive exposure to digital learning among medical students has been increasingly reported, and prolonged use of electronic devices in educational settings can be expected to lead to sustained forward head posture, postural overload, and musculoskeletal strain. Recent reports emphasize the increasing dependence on screen-based learning modes in medical education and point to concerns over their unintended ergonomic consequences. In this regard, structured small-group modules, team-based learning, problem-based learning, flipped classroom approaches with reduced real-time device dependence, and discussion-oriented sessions could be used to minimize excessive digital exposure. Embedding such models into pedagogy may reduce continuous screen use, foster varied postures, and have the potential to reduce the incidence of posture-related musculoskeletal issues among medical students without compromise to educational outcomes [24,25].

There are some limitations in this study that must be considered. The cross-sectional design of the study limits the causal inference between the use of a mobile phone and text neck syndrome. Purposive sampling may introduce selection bias and reduce generalizability. No multivariable regression analysis was conducted because only one variable showed a statistically significant association in the bivariate analysis; this limits adjustments for potential confounders. Besides, reliance on self-reported measures may lead to recall or reporting bias.

## Conclusions

The present study demonstrated that undergraduate medical students had a very high prevalence of text neck syndrome, and a significant proportion had mild to moderate neck disability. Lower awareness about musculoskeletal problems related to the use of mobile phones was significantly associated with higher neck disability. The results emphasize the necessity of institution-based awareness and posture-oriented educational interventions for medical students. Longitudinal studies are needed to clarify the causal pathways better and the strategies for prevention.
